# THE M. POWELL LAWTON AWARD LECTURE AND PRESENTATION

**DOI:** 10.1093/geroni/igad104.1620

**Published:** 2023-12-21

**Authors:** Tiffany Washington

## Abstract

The M. Powell Lawton Award Lecture will feature an address by the 2022 recipient Jon Pynoos, PhD, FGSA, FAGHE, of the University of Southern California. This session will also include the presentation of the 2023 M. Powell Lawton Award to recipient. The M. Powell Lawton Award is presented annually to an individual who has made outstanding contributions from applied research that has benefited older people and their care. The Lawton Award is generously funded by the Polisher Research Institute of Abramson Senior Care.

